# Hindered aryl bromides for regioselective palladium-catalysed direct arylation at less favourable C5-carbon of 3-substituted thiophenes

**DOI:** 10.3762/bjoc.10.123

**Published:** 2014-05-27

**Authors:** Rongwei Jin, Charles Beromeo Bheeter, Henri Doucet

**Affiliations:** 1 Institut Sciences Chimiques de Rennes, UMR 6226 CNRS-Université de Rennes "Organometalliques, Material and Catalysis", Campus de Beaulieu, 35042 Rennes, France. Fax: +33 (0)2-23-23-69-39; Tel: +33 (0)2-23-23-63-84

**Keywords:** aryl bromides, atom economy, C–H bond activation, palladium, regioselectivity, thiophenes

## Abstract

The use of the congested aryl bromide 2-bromo-1,3-dichlorobenzene as coupling partner allows to modify the regioselectivity of the arylation of 3-substituted thiophene derivatives in favour of carbon C5. The coupling of this aryl bromide with a variety of 3-substituted thiophenes gave in all cases the desired 5-arylation products in moderate to good yields using only 0.5 mol % of a phosphine-free and air-stable palladium catalyst. Then, from these 5-arylthiophenes, a second palladium-catalysed C–H bond functionalization at C2 of the thiophene ring allows the synthesis of 2,5-diarylthiophenes with two different aryl units.

## Introduction

As thiophenes bearing aryl substituents are known to be present in several bioactive molecules and are used as precursors of materials, the regioselective introduction of aryls on thiophenes is an important research area in organic synthesis. The coupling of thiophene derivatives with aryl halides via a C–H bond activation/functionalisation [1–12] provides an environmentally attractive and cost-effective procedure for the preparation of a variety of arylated thiophenes. For such coupling reactions, the major byproduct is a base associated to HX, instead of metallic salts which are produced under the more classical Negishi, Suzuki or Stille cross-coupling reactions. Moreover, direct arylation avoids the preliminary preparation of organometallics reducing the number of steps to prepare these arylthiophenes.

**Scheme 1:**
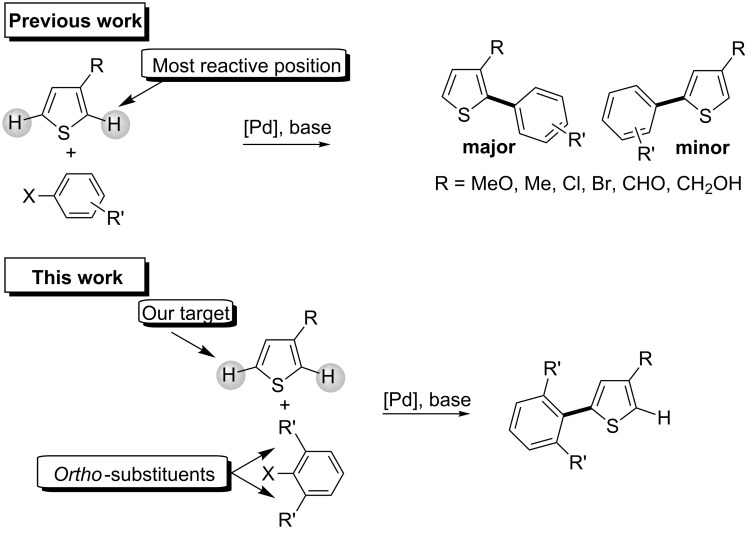
Regioselectivity of coupling reactions of 3-substituted thiophenes with aryl halides.

The regioselective arylation via a Pd-catalysed C–H bond activation at carbon C5 of 2-substituted thiophenes has been largely described in recent years [13–22]. On the other hand, the Pd-catalysed direct arylation of 3-substituted thiophenes has attracted much less attention (Scheme 1, top) [23–31]. With such thiophene derivatives, in most cases, either C2-arylated thiophenes or mixtures of C2- and C5-arylated products have been obtained. For example, Sharp et al. described conditions for the regioselective arylation at carbons C2 or C5 of methyl 3-thiophenecarboxylate [23]. The reaction performed with Pd(PPh_3_)_4_ as the catalyst in toluene led selectively to the 2-arylated thiophene; whereas, the use of Pd_2_(dba)_3_ in NMP afforded a mixture of 2- and 5-arylated thiophenes in a 15:51 ratio. Lemaire and co-workers have reported the C2-arylation of 3-formyl-, 3-cyano- and 3-nitrothiophenes with iodobenzenes [24–25]. Forgione, Bilodeau et al. reported that the reaction of 3-methylthiophene with bromobenzene using Pd[(P(*t*-Bu)_3_]_2_ as the catalyst affords a mixture of 2- and 5-phenylated thiophenes in a 3.3:1 ratio [26]. Fagnou and co-workers reported that the coupling of 3-*n*-hexylthiophene with 4-bromonitrobenzene also led to a mixture of C2- and C5-arylation products in a 1.3:1 ratio [27]. Then, they blocked one position on the thiophene ring using a chloro-substituent in order to selectively arylate positions C2 or C5. The direct arylation of 3-methoxythiophene, which was studied by Borghese and co-workers afforded regioselectively the C2-arylated thiophenes in moderate to high yields [28]. Finally, in the course of their studies on sp^3^ C–H bond activation, Baudoin and Pierre recently reported that in the presence of an extremely bulky substituent at C3 of a thiophene derivative, the C5-arylated compounds were selectively obtained in good yields [29]. In summary, due to the presence of two reactive C–H bonds in 3-substituted thiophenes (with position C2 generally slightly more reactive than position C5), the control of the regioselectivity of the palladium-catalysed direct arylation of such thiophene derivatives especially to provide 5-arylthiophenes remains a challenging reaction.

Our goal was to promote arylation at carbon C5 of a range of 3-substituted thiophenes without the use of a blocking group at carbon C2. To our knowledge, *ortho*-substituents on aryl bromides have not been employed as directing groups for palladium-catalysed direct arylation of 3-substituted thiophenes. The use of congested aryl bromides for such couplings would certainly modify the regioselectivity in favour of arylation at the less hindered thiophene position. Herein, we wish to report on the influence of such *ortho*-substituents on aryl bromides on the regioselectivity of the palladium-catalysed direct arylations of 3-substituted thiophenes.

## Results and Discussion

**Scheme 2:**
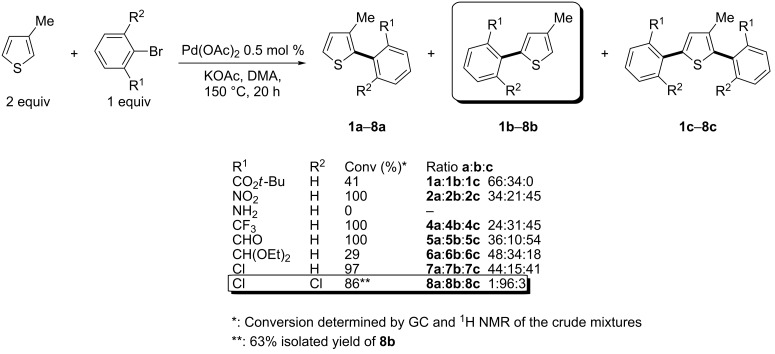
Regioselectivity of the arylation of 2-methylthiophene with *ortho*-substituted aryl bromides.

We first studied the palladium-catalysed direct arylation of 3-methylthiophene using *tert*-butyl 2-bromobenzoate as the coupling partner (Scheme 2). Based on previous results [19], DMA was initially chosen as the solvent and KOAc as the base. The reactions were performed at 150 °C under argon in the presence of 0.5 mol % Pd(OAc)_2_ as the catalyst. However, under these conditions, a poor regioselectivity was observed as the desired C5-arylation product **1b** was only obtained in 34% selectivity together with 66% of C2-arylation product **1a**. Moreover, a moderate conversion of this aryl bromide was observed and purification by silica gel chromatography afforded a mixture of **1a** and **1b**. A slightly lower selectivity in favour of the formation of C5-arylation product **2b** was observed using 2-bromonitrobenzene as the coupling partner, as a large amount of 2,5-diarylated product **2c** was also produced with a **2a**:**2b**:**2c** ratio of 34:21:45. However, a complete conversion of 2-bromonitrobenzene was observed. 2-Bromoaniline was found to be unreactive and was recovered. It should be noted that no amination reaction of 2-bromoaniline due to self-coupling was observed. From 2-(trifluoromethyl)bromobenzene, a very similar mixture of regioisomers than with 2-bromonitrobenzene was obtained, as **4a**, **4b** and **4c** were formed in a 24:31:45 ratio. The use of 2-bromobenzaldehyde was not successful, as the desired product **5b** was only obtained with 10% selectivity. From more hindered 2-bromobenzaldehyde diethyl acetal, the selectivity in favour of desired product **6b** was slightly higher (34%), although still not synthetically useful. Next, we employed 2-chlorobromobenzene as the coupling partner. However, again the desired product **7b** was only formed with 15% selectivity. It should be noted that with these aryl bromides, in all cases, mixtures of regioisomers **a** and **b** were obtained after column chromatography. On the other hand, the use of 2-bromo-1,3-dichlorobenzene allowed to obtain very selectively the desired 5-arylation product **8b**. Only traces of the C2-arylated thiophene **8a** and a low amount of 2,5-diarylated product **8c** were detected by ^1^H NMR and GC–MS analysis of the crude mixture. Moreover a high conversion of 86% of this aryl bromide was observed using only 0.5 mol % Pd(OAc)_2_ as the catalyst.

**Scheme 3:**
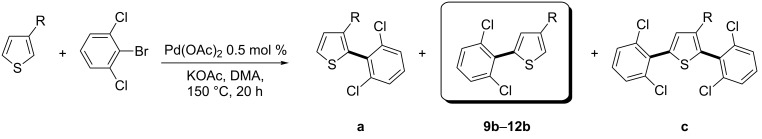
Direct arylation of 3-substituted thiophenes with 2-bromo-1,3-dichlorobenzene.

Then, we studied the scope of the coupling of 2-bromo-1,3-dichlorobenzene, using other 3-substituted thiophene derivatives (Scheme 3, Table 1). Both 3-chlorothiophene and ethyl thiophene-3-carboxylate led to the desired 5-arylation products **9b** and **10b** with 89% selectivity and in 44% and 65% yields, respectively (Table 1, entries 1 and 2). A slightly higher regioselectivity in favour of the C5-arylation was observed in the presence of 3-acetylthiophene, as **11b** was obtained with 92% selectivity (Table 1, entry 3). From ethyl thiophen-3-ylacetate, **12b** was formed with 90% selectivity and in 62% yield (Table 1, entry 4). On the other hand, from methyl (*E*)-3-thiophen-3-ylacrylate, 3-bromothiophene or 3-formylthiophene, mixtures of unidentified products were obtained.

**Table 1:** Direct arylation of 3-substituted thiophenes with 2-bromo-1,3-dichlorobenzene (Scheme 3).^a^

Entry	Heteroarene	Major product	Ratio **a**:**b**:**c**	Yield^b^ of regioisomer **b** (%)

1	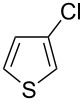	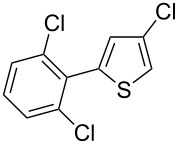 **9b**	3:89:8	44
2	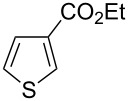	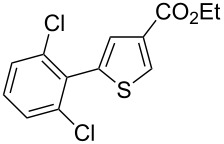 **10b**	3:89:8	65
3	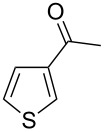	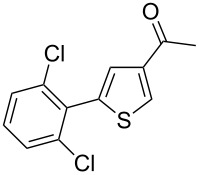 **11b**	0:92:8	57
4	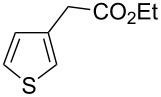	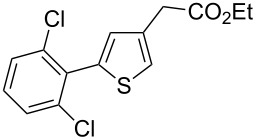 **12b**	2:90:8	62

^a^Conditions: Pd(OAc)_2_ (0.5 mol %), aryl bromide (1 mmol), thiophene derivative (2 mmol), KOAc (2 mmol), DMA (3 mL), 150 °C, 20 h; ^b^isolated yields of regioisomers **9b**–**12b**.

**Scheme 4:**
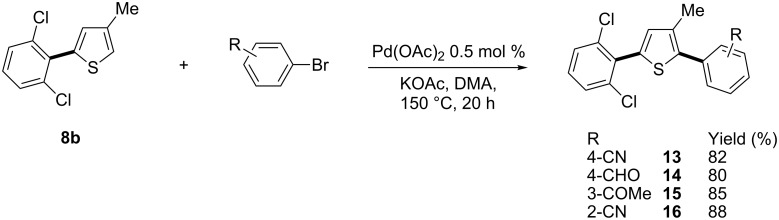
Pd-catalysed C2-arylation of **8b** with aryl bromides.

The reactivity for arylation at carbon C2 of the previously prepared 2-(2,6-dichlorophenyl)-4-methylthiophene (**8b**), via a second Pd-catalysed C–H bond activation, was also investigated (Scheme 4). 4-Bromobenzonitrile and **8b** in the presence of 0.5 mol % Pd(OAc)_2_ and KOAc as the base gave the desired coupling product **13** in 82% yield. A similar reactivity was observed with 4-bromobenzaldehyde and 3-bromoacetophenone affording **14** and **15** in 80% and 85% yields, respectively. Finally, more congested 2-bromobenzonitrile was reacted with **8b** to provide **16** in 88% yield. These regioselective sequential arylations of a 3-substituted thiophene offer a simple access to a variety of 2,5-diarylthiophenes with two different aryl units.

## Conclusion

In summary, we have demonstrated that the use of the congested coupling partner 2-bromo-1,3-dichlorobenzene allows to direct the arylation to the unfavourable C5 position of 3-substituted thiophenes. These less favoured regioisomers can be selectively obtained in moderate to good yields using a range of 3-substituted thiophenes, as chloro, ester, acetyl or ethyl acetate substituents are tolerated. Moreover, the sequential catalytic C5 and C2 arylations, allow the preparation of 2,5-diarylthiophenes with two different aryl units in two steps. The major byproduct of these couplings is KBr/AcOH instead of metallic salts as with more classical coupling procedures. For these reasons, this process gives an economically viable access to C5-arylated 3-substituted heteroaromatics.

## Experimental

### General

All reactions were perfomed in Schlenk tubes under argon. DMA analytical grade was not distilled before use. Commercial aryl bromide derivatives were used without purification. ^1^H NMR (400 MHz), ^13^C NMR (100 MHz) spectra were recorded in CDCl_3_ solutions on Bruker GPX (400 MHz). GC was performed on a Shimadzu GC-2014 and GC–MS was performed on a Shimadzu QP-2010S. Chemical shifts are reported in ppm relative to CDCl_3_ (^1^H: 7.26 and ^13^C: 77.0). Flash chromatography was performed on silica gel (230–400 mesh).

#### General procedure for direct arylations

The aryl bromide (1 mmol), thiophene derivative (2 mmol), KOAc (2 mmol), Pd(OAc)_2_ (0.005 mmol, 1.1 mg) and DMA (3 mL) were introduced in a Schlenk tube under argon equipped with a magnetic stirring bar. The Schlenk tube was placed in an oil bath pre-heated at 150 °C, and the reaction mixture was allowed to stir for 20 h. After cooling, the crude reaction mixture was analysed by gas chromatography and ^1^H NMR to determine the conversion of the aryl bromide and the regioselectivity of the arylation. The solvent was removed by heating under vacuum, then the residue was charged onto a silica gel column.

***tert*-Butyl 2-(4-methylthiophen-2-yl)benzoate (1b):** After column chromatography (pentane/diethyl ether 98:2), a mixture of products **1a** and **1b** was obtained in 24% (0.066 g) yield. ^1^H NMR (400 MHz, CDCl_3_) δ 6.84 (s, 1H), 2.20 (s, 3H). **1a** was also observed: ^1^H NMR (400 MHz, CDCl_3_) δ 7.14 (d, *J* = 5.0 Hz, 1H), 6.81 (d, *J* = 5.0 Hz, 1H), 1.97 (s, 3H).

**4-Methyl-2-(2-nitrophenyl)thiophene (2b):** After column chromatography (pentane/diethyl ether 95:5), a mixture of products **2a** and **2b** was obtained in 60% (0.131 g) yield. ^1^H NMR (400 MHz, CDCl_3_) δ 7.63 (d, *J* = 8.0 Hz, 1H), 6.91 (s, 1H), 2.19 (s, 3H). **2a** was also observed: ^1^H NMR (400 MHz, CDCl_3_) δ 7.83 (d, *J* = 8.0 Hz, 1H), 7.22 (d, *J* = 5.0 Hz, 1H), 6.83 (d, *J* = 5.0 Hz, 1H), 1.99 (s, 3H).

**4-Methyl-2-(2-trifluoromethylphenyl)thiophene (4b):** After column chromatography (pentane/diethyl ether 90:10), a mixture of products **4a**, **4b** and **4c** was obtained in 66% (0.160 g) yield. ^1^H NMR (400 MHz, CDCl_3_) δ 6.88 (s, 1H), 6.83 (s, 1H), 1.95 (s, 3H). **4a** was also observed: ^1^H NMR (400 MHz, CDCl_3_) δ 7.20 (d, *J* = 5.1 Hz, 1H), 6.81 (d, *J* = 5.1 Hz, 1H). **4c** was also observed: ^1^H NMR (400 MHz, CDCl_3_) δ 7.75–7.65 (m, 2H), 7.55–7.35 (m, 6H), 6.82 (s, 1H), 1.95 (s, 3H).

**2-(4-Methylthiophen-2-yl)benzaldehyde (5b):** After column chromatography (pentane/diethyl ether 95:5), a mixture of products **5a** and **5b** was obtained in 51% (0.103 g) yield. ^1^H NMR (400 MHz, CDCl_3_) δ 10.14 (s, 1H), 7.92 (d, *J* = 7.8 Hz, 1H), 7.60–7.35 (m, 3H), 6.97 (s, 1H), 6.81 (s, 1H), 2.25 (s, 3H). **5a** was also observed: ^1^H NMR (400 MHz, CDCl_3_) δ 9.85 (s, 1H), 7.95 (d, *J* = 7.6 Hz, 1H), 7.56 (t, *J* = 7.6 Hz, 1H), 7.44 (t, *J* = 7.6 Hz, 1H), 7.36 (d, *J* = 7.6 Hz, 1H), 7.27 (d, *J* = 5.0 Hz, 1H), 6.89 (d, *J* = 5.0 Hz, 1H), 2.02 (s, 3H).

**2-(2-Chlorophenyl)-4-methylthiophene (7b):** After column chromatography (pentane/diethyl ether 95:5), a mixture of products **7a** and **7b** was obtained in 64% (0.133 g) yield. ^1^H NMR (400 MHz, CDCl_3_) δ 7.11 (s, 1H), 6.90 (s, 1H), 2.24 (s, 3H). **7a** was also observed: ^1^H NMR (400 MHz, CDCl_3_) δ 6.87 (d, *J* = 5.0 Hz, 1H), 2.04 (s, 3H).

**2-(2,6-Dichlorophenyl)-4-methylthiophene (8b):** From 2-bromo-1,3-dichlorobenzene (0.226 g, 1 mmol) and 3-methylthiophene (0.196 g, 2 mmol), **8b** was obtained in 63% (0.153 g) yield as an oil after column chromatography (pentane). ^1^H NMR (400 MHz, CDCl_3_) δ 7.31 (d, *J* = 7.5 Hz, 2H), 7.15 (t, *J* = 7.5 Hz, 1H), 6.98 (s, 1H), 6.75 (s, 1H), 2.25 (s, 3H); ^13^C NMR (50 MHz, CDCl_3_) δ 137.4, 136.6, 136.4, 133.1, 130.8, 129.7, 128.0, 122.1, 15.8; Anal. calcd for C_11_H_8_Cl_2_S (243.15): C, 54.34; H, 3.32; found: C, 54.19; H, 3.17. Traces of **8a** and **8c** were also detected by GC–MS analysis of the crude mixture.

**4-Chloro-2-(2,6-dichlorophenyl)thiophene (9b):** From 2-bromo-1,3-dichlorobenzene (0.226 g, 1 mmol) and 3-chlorothiophene (0.237 g, 2 mmol), **9b** was obtained in 44% (0.116 g) yield as an oil after column chromatography (pentane). ^1^H NMR (400 MHz, CDCl_3_) δ 7.32 (d, *J* = 7.5 Hz, 2H), 7.23–7.17 (m, 2H), 6.83 (d, *J* = 1.2 Hz, 1H); ^13^C NMR (50 MHz, CDCl_3_) δ 137.4, 136.5, 131.7, 130.4, 128.8, 128.2, 124.9, 121.4; Anal. calcd for C_10_H_5_Cl_3_S (263.57): C, 45.57; H, 1.91; found: C, 45.67; H, 1.90. Traces of **9a** and **9c** were also detected by GC–MS analysis of the crude mixture.

**Ethyl 5-(2,6-dichlorophenyl)thiophene-3-carboxylate (10b):** From 2-bromo-1,3-dichlorobenzene (0.226 g, 1 mmol) and ethyl thiophene-3-carboxylate (0.312 g, 2 mmol), **10b** was obtained in 65% (0.196 g) yield as an oil after column chromatography (pentane/diethyl ether 98:2). ^1^H NMR (400 MHz, CDCl_3_) δ 8.16 (s, 1H), 7.37 (d, *J* = 7.5 Hz, 2H), 7.34 (s, 1H), 7.22 (t, *J* = 7.5 Hz, 1H), 4.29 (q, *J* = 7.5 Hz, 2H), 1.31 (t, *J* = 7.5 Hz, 3H); ^13^C NMR (50 MHz, CDCl_3_) δ 162.6, 137.3, 136.6, 133.8, 133.7, 131.8, 130.3, 129.3, 128.2, 60.8, 14.3; Anal. calcd for C_13_H_10_Cl_2_O_2_S (301.19): C, 51.84; H, 3.35; found: C, 51.99; H, 3.17. Traces of **10a** and **10c** were also detected by GC–MS analysis of the crude mixture.

**1-[5-(2,6-Dichlorophenyl)thiophen-3-yl]ethanone (11b):** From 2-bromo-1,3-dichlorobenzene (0.226 g, 1 mmol) and 1-thiophen-3-ylethanone (0.252 g, 2 mmol), **11b** was obtained in 57% (0.154 g) yield as an oil after column chromatography (pentane/diethyl ether 95:5). ^1^H NMR (400 MHz, CDCl_3_) δ 8.10 (s, 1H), 7.37 (d, *J* = 7.5 Hz, 2H), 7.34 (s, 1H), 7.22 (t, *J* = 7.5 Hz, 1H), 2.49 (s, 3H); ^13^C NMR (50 MHz, CDCl_3_) δ 191.0, 141.3, 136.7, 135.4, 132.5, 130.6, 129.4, 127.4, 127.2, 26.4; Anal. calcd (%) for C_12_H_8_Cl_2_OS (271.16): C, 53.15; H, 2.97; found: C, 53.31; H, 3.07. Traces of **11c** were also detected by GC–MS analysis of the crude mixture.

**Ethyl [5-(2,6-dichlorophenyl)thiophen-3-yl]acetate (12b):** From 2-bromo-1,3-dichlorobenzene (0.226 g, 1 mmol) and ethyl thiophen-3-ylacetate (0.340 g, 2 mmol), **12b** was obtained in 62% (0.195 g) yield as an oil after column chromatography (pentane/diethyl ether 95:5). ^1^H NMR (400 MHz, CDCl_3_) δ 7.32 (d, *J* = 7.5 Hz, 2H), 7.22 (s, 1H), 7.16 (t, *J* = 7.5 Hz, 1H), 6.89 (s, 1H), 4.12 (q, *J* = 7.5 Hz, 2H), 3.60 (s, 2H), 1.19 (t, *J* = 7.5 Hz, 3H); ^13^C NMR (50 MHz, CDCl_3_) δ 170.9, 136.7, 136.6, 133.5, 132.8, 130.2, 129.9, 128.1, 124.2, 60.9, 36.2, 14.2; Anal. calcd for C_14_H_12_Cl_2_O_2_S (315.22): C, 53.34; H, 3.84; found: C, 53.21; H, 3.70. Traces of **12a** and **12c** were also detected by GC–MS analysis of the crude mixture.

**4-[5-(2,6-Dichlorophenyl)-3-methylthiophen-2-yl]benzonitrile (13):** From 4-bromobenzonitrile (0.182 g, 1 mmol) and 2-(2,6-dichlorophenyl)-4-methylthiophene (**8b**, 0.486 g, 2 mmol), **13** was obtained in 82% (0.282 g) yield as an oil after column chromatography (pentane/diethyl ether 95:5). ^1^H NMR (400 MHz, CDCl_3_) δ 7.63 (d, *J* = 7.5 Hz, 2H), 7.56 (d, *J* = 7.5 Hz, 2H), 7.34 (d, *J* = 7.5 Hz, 2H), 7.19 (t, *J* = 7.5 Hz, 1H), 6.80 (s, 1H), 2.33 (s, 3H); ^13^C NMR (50 MHz, CDCl_3_) δ 139.1, 137.2, 136.4, 136.3, 134.7, 133.2, 132.3, 132.2, 130.0, 129.2, 128.2, 118.8, 110.6, 15.8; Anal. calcd for C_18_H_11_Cl_2_NS (344.26): C, 62.80; H, 3.22; found: C, 63.04; H, 3.17.

**4-(5-(2,6-Dichlorophenyl)-3-methylthiophen-2-yl)benzaldehyde (14):** From 4-bromobenzaldehyde (0.185 g, 1 mmol) and 2-(2,6-dichlorophenyl)-4-methylthiophene (**8b**, 0.486 g, 2 mmol), **16** was obtained in 80% (0.278 g) yield as an oil after column chromatography (pentane/diethyl ether 85:15). ^1^H NMR (400 MHz, CDCl_3_) δ 9.97 (s, 1H), 7.86 (d, *J* = 7.5 Hz, 2H), 7.63 (d, *J* = 7.5 Hz, 2H), 7.34 (d, *J* = 7.5 Hz, 2H), 7.19 (t, *J* = 7.5 Hz, 1H), 6.81 (s, 1H), 2.36 (s, 3H); ^13^C NMR (50 MHz, CDCl_3_) δ 191.6, 140.6, 137.9, 136.4, 136.1, 134.8, 134.6, 133.2, 132.3, 130.0, 129.1, 128.2, 15.5; Anal. calcd for C_18_H_12_Cl_2_OS (347.26): C, 62.26; H, 3.48; found: C, 62.09; H, 3.50.

**1-{3-[5-(2,6-Dichlorophenyl)-3-methylthiophen-2-yl]phenyl}ethanone (15):** From 3-bromoacetophenone (0.199 g, 1 mmol) and 2-(2,6-dichlorophenyl)-4-methylthiophene (**8b**, 0.486 g, 2 mmol), **15** was obtained in 85% (0.307 g) yield as a white solid after column chromatography pentane/diethyl ether 85:15). ^1^H NMR (400 MHz, CDCl_3_) δ 8.04 (s, 1H), 7.84 (d, *J* = 7.5 Hz, 1H), 7.65 (d, *J* = 7.5 Hz, 1H), 7.45 (t, *J* = 7.5 Hz, 1H), 7.34 (d, *J* = 7.5 Hz, 2H), 7.17 (t, *J* = 7.5 Hz, 1H), 6.79 (s, 1H), 2.58 (s, 3H), 2.31 (s, 3H); ^13^C NMR (50 MHz, CDCl_3_) δ 196.8, 137.1, 136.4, 135.5, 134.0, 133.9, 132.5, 132.4, 131.7, 131.6, 128.8, 127.9, 127.8, 127.1, 126.1, 25.7, 14.1; Anal. calcd for C_19_H_14_Cl_2_OS (361.29): C, 63.19; H, 3.91; found: C, 63.04; H, 3.99.

**2-[5-(2,6-Dichlorophenyl)-3-methylthiophen-2-yl]benzonitrile (16):** From 2-bromobenzonitrile (0.182 g, 1 mmol) and 2-(2,6-dichlorophenyl)-4-methylthiophene (**8b**, 0.486 g, 2 mmol), **16** was obtained in 88% (0.303 g) yield as an oil after column chromatography (pentane/diethyl ether 95:5). ^1^H NMR (400 MHz, CDCl_3_) δ 7.77 (d, *J* = 7.8 Hz, 1H), 7.63 (t, *J* = 7.8 Hz, 1H), 7.57 (d, *J* = 7.8 Hz, 1H), 7.46 (t, *J* = 7.8 Hz, 1H), 7.40 (d, *J* = 7.8 Hz, 2H), 7.24 (t, *J* = 7.8 Hz, 1H), 6.88 (s, 1H), 2.27 (s, 3H); ^13^C NMR (50 MHz, CDCl_3_) δ 136.9, 135.7, 135.6, 135.1, 133.5, 132.4, 131.4, 131.3, 130.9, 130.8, 128.9, 127.2, 127.1, 117.0, 112.8, 13.9; Anal. calcd for C_18_H_11_Cl_2_NS (344.26): C, 62.80; H, 3.22; found: C, 63.01; H, 3.40.
